# 563. *Mycoplasma genitalium* with Fluoroquinolone Resistance: Design and Development of a Multiplex Real-Time PCR Assay for Detection and Differentiation

**DOI:** 10.1093/ofid/ofad500.632

**Published:** 2023-11-27

**Authors:** Boris Alabyev, Irina Ankoudinova, Noah Scarr, Becca Decker, Eugene Lukhtanov

**Affiliations:** ElitechGroup MDX LLC, Bothell, Washington; ElitechGroup MDX LLC, Bothell, Washington; Elitech Group MDx, Bothell, Washington; ElitechGroup MDX LLC, Bothell, Washington; ElitechGroup MDX LLC, Bothell, Washington

## Abstract

**Background:**

*Mycoplasma genitalium* is a sexually transmitted bacterium causing urethritis in men and associated with cervicitis and pelvic inflammatory disease in women. Only a few antimicrobial classes have activity against mycoplasmas. The first-line treatment for M. genitalium infections is doxycycline followed by azithromycin. Due to overuse of azithromycin to treat infections, resistance to azithromycin has been rapidly increasing. The moxifloxacin of fluoroquinolones family of antibiotics was 100% effective against M. genitalium, but recently the resistance markers for fluoroquinolone treatment have also emerged. Multiple studies attribute fluoroquinolone treatment failure to single nucleotide polymorphisms (SNPs) in parC and gyrA genes.

We aim to demonstrate proof of principle for a Real-Time PCR assay that simultaneously detects *M. genitalium* and fluoroquinolone resistance.

**Methods:**

The MGB Alert M. genitalium with fluoroquinolone resistance RUO Detection Reagent is a multiplex of real-time PCR reagents that simultaneously detect *M. genitalium* DNA and distinguish fluoroquinolone resistance-associated mutations and wild type in *M. genitalium*. The assay targets parC and gyrA genes and includes a set of two primers and two probes specific to *M. genitalium*. The **DSQ** probe identifies the species DNA. The **Pleiades** hybridization probes serve to identify and distinguish a wild type genotype and several known resistance-conferring point mutations by melt curve analysis.

**Results:**

Analytical Study:

Analytical sensitivity was determined and verified at 1.42 copy of bacterial genome per reaction.

A linear range was established from 1e7 to 10 genome copies per reaction.

Test results showed no cross-reactivity with organisms that might be present in normal vaginal swabs and urine specimens.

**Conclusion:**

Preliminary analytical evaluation of the MGB Alert M. genitalium with fluoroquinolone resistance RUO Detection Reagent indicates potential as a valuable tool in the diagnosis of *M. genitalium* and resistance guided treatment.

**Disclosures:**

**Boris Alabyev, PhD**, ELITechGroup MDx LLC: Salary **Irina Ankoudinova, MS**, ELITechGroup MDx LLC: Salary **Noah Scarr, BS**, ELITechGroup MDx LLC: Salary **Becca Decker, BS**, ELITechGroup MDx LLC: Salary **Eugene Lukhtanov, PhD**, ELITechGroup MDx LLC: Salary

